# Transglutaminase-Crosslinked Collagen Gel Delays Gastrointestinal Transit and Prolongs Serum Appearance of Antioxidant Collagen Di- and Tripeptides in Mice

**DOI:** 10.3390/nu18213602

**Published:** 2026-10-30

**Authors:** Yapeng Lu, Ying He, Xin Feng, Mi Yan, Yexing Liang, Liang Ma, Yuhao Zhang

**Affiliations:** 1 College of Food Science, Southwest University, Chongqing 400715, China; 2 Chongqing Academy of Agricultural Sciences, Chongqing 401329, China

**Keywords:** transglutaminase-crosslinked collagen gel, gastrointestinal transit, collagen dipeptides and tripeptides, antioxidants

## Abstract

Background: Transglutaminase-crosslinked collagen gel (CHG) is a collagen product cross-linked with transglutaminase (TGase) that exhibits good thermal stability and has potential applications in functional food formulations. However, it remains unclear whether the gelation of CHG alters the metabolic fate of collagen-derived bioactive peptides in the gastrointestinal tract and their distribution within the body. Methods: This study investigated the retention of CHG in the gastrointestinal tract of male C57BL/6J mice, as well as its serum peptide levels and antioxidant activity. Results: Compared with unhydrolyzed (CNS) and hydrolyzed collagen (CHS) sols, CHG significantly prolonged gastric retention and slowed intestinal transit, while gastric contents exhibited larger particle sizes and a more sustained release of protein. The CHG also increased the expression of genes associated with Cajal interstitial (ICC) cells in the gastric antrum (HCN1, Trpm7, CX43, and c-kit) and prolonged the secretion of leptin, cholecystokinin, serotonin, and vasoactive intestinal peptide. Serum peptide analysis showed that the concentrations of most collagen dipeptides and tripeptides, peaked between 0.5 and 1.5 h and then gradually declined; notably, the concentration of Pro-Hyp in the CHG group remained higher than that in the control group at 3 h, indicating prolonged serum appearance. Serum peptides from CHG group (particularly Pro-Hyp) exhibited total antioxidant capacity and hydroxyl radical scavenging activity, and the mixed-peptide solution significantly reduced the accumulation of reactive oxygen species in H_2_O_2_-induced skin fibroblasts (*p* < 0.001). Conclusions: These findings suggest that the cross-linked structure of CHG alters gastrointestinal metabolic processes and is associated with the persistent presence of collagen-derived dipeptides and tripeptides in serum.

## 1. Introduction

Collagen is a structural protein found in abundance in mammals, accounting for approximately 30 percent of the body’s total protein, and is widely distributed in the skin, bones, tendons, and cartilage [1,2]. Oral administration of collagen hydrolysate leads to an increase in the levels of hydroxyproline (Hyp) dipeptides and tripeptides in serum; these peptides have been shown to possess various biological activities, including antioxidant and skin-protective effects [3,4]. However, collagen hydrolysates generally have poor thermal stability, which limits their application in thermally processed foods. Enzymatic cross-linking using transglutaminase (TGase) can improve their thermal stability and form a three-dimensional gel network, thereby producing TGase-crosslinked collagen gel (CHG) [5].

The physical structure of ingested protein has a significant impact on its behavior in the gastrointestinal tract. Although it is generally believed that protein cross-linking reduces digestibility, recent evidence suggests that moderate TGase cross-linking can promote the release of peptides during gastric digestion and increase the variety of collagen peptides detected in serum following absorption [6,7]. This means that the physical structure of collagen determines its metabolic process in the gastrointestinal tract.

However, to truly reveal the unique value of CHG, it is essential to address a key question: what exactly happens during the digestion of CHG in the body? This process involves two closely related stages. The first is the gastrointestinal transit process, specifically the kinetics of CHG’s disintegration, changes in particle size, and passage into the intestine under the influence of mechanical peristalsis and pepsin in the stomach. The second is the effect of CHG on serum levels of collagen dipeptides and tripeptides. Specifically, the therapeutic efficacy of oral CHG depends to a large extent on the specific peptide sequences in the blood; however, it remains unclear how the levels of collagen dipeptides and tripeptides in serum change following the digestion of CHG.

Gastrointestinal digestion is not a passive physical process, but is precisely regulated by the pacemaker potentials of Cajal interstitial cells (ICCs) in the gastric antrum and by the neuroendocrine networks of the brain–gut axis (such as LEP, CCK, and the neurotransmitters 5-HT and VIP) [8,9]. The ICCs are pacemaker and signaling cells in the gastrointestinal tract that regulate the rhythmic contractions of the gastric smooth muscle by generating slow-wave potentials, thereby directly influencing gastric retention [10]. Studies have shown that the maintenance of ICC function depends on the coordinated expression of various ion channels and signaling proteins. Although previous studies have confirmed that the physical properties of protein-digested food (such as particle size, viscosity, and gel strength) can regulate gastric antral contractions and the expression of genes related to gastrointestinal motility [11], it remains unclear how the physical gel structure of CHG, which persists in the stomach, differentially activates these upstream regulatory signals. More importantly, elucidating the upstream regulatory mechanisms of CHG-induced gastric retention will help us gain a deeper understanding of the underlying biological basis for its ability to delay gastric retention time.

Although our previous study demonstrated that TGase cross-linking alters the digestion products of collagen gel and the serum peptide profile, changes in serum collagen dipeptides and tripeptides were not analyzed due to the specific nature of the detection methods [7]. Furthermore, questions regarding how the structure of cross-linked gel regulates gastrointestinal transit and the timing of the appearance of specific collagen dipeptides and tripeptides remain unanswered. Therefore, this study focused on the gastric retention and intestinal transit behavior of CHG, the involvement of ICCs and brain–gut axis signaling, and the antioxidant activity of specific collagen dipeptides and tripeptides in vivo. Specifically, we tracked gastric retention and intestinal transit using fluorescence labeling, examined ICC-associated gene expression and brain–gut hormone secretion, and quantified serum collagen di-/tripeptides by LC-MS/MS. We further evaluated the antioxidant activity of the major absorbed peptides in vitro and in H_2_O_2_-induced oxidative damage in skin fibroblasts. This study aims to provide further in vivo evidence to support the development of CHG as a novel functional food.

## 2. Materials and Methods

### 2.1. Experimental Materials

Defatted pork skin (food grade) was provided by Henan Pinfen Trading Co., Ltd. (Zhengzhou, Henan, China). Papain (biochemical grade) was purchased from Beijing Suolebao Technology Co., Ltd. (Beijing, China). Glutamyltransferase (TGase, 200 U/g) was supplied by Shanghai Foodchem Chemical Co., Ltd. (Shanghai, China). Sucrose fatty acid esters (analytical grade) were purchased from Shanghai Aladdin Biochemical Technology Co., Ltd. (Shanghai, China). NaCl (analytical grade) was obtained from Chengdu Kelong Chemicals Co., Ltd. (Chengdu, Sichuan, China). Gly-Pro, Hyp-Gly, Pro-Hyp, Glu-Hyp, Ser-Hyp-Gly, and Gly-Pro-Hyp were purchased from Shanghai Chutai Biotechnology Co., Ltd. (Shanghai, China; purity ≥ 98%). Enzyme-linked immunosorbent assay (ELISA) kits for murine leptin (LEP), glucagon-like peptide-1 (GLP-1), cholecystokinin (CCK), serotonin (5-HT), and vasoactive intestinal peptide (VIP) were all purchased from Jiangsu ELISA Biotechnology Co., Ltd. (Nanjing, Jiangsu, China). The mRNA extraction kit (TIANGEN, DP451) was supplied by Tiangen Biotechnology. The quantitative PCR reverse transcription kit (abm, Cat#G592) was purchased from Applied Biological Materials Inc. (abm). The real-time fluorescent quantitative PCR kit (E096-01A) was provided by Novoprotein Technologies. All primers used for RT-qPCR detection of HCN1, Trpm7, CX43, c-kit, and β-actin were obtained from Sangon Biotech Co., Ltd. (Shanghai, China) The primer sequences are listed in Table 1.

### 2.2. Preparation of Collagen Gel Samples

Fresh pork skin was obtained, and the subcutaneous fat was removed. The skin was washed and cut into rectangular pieces. The pieces were placed in a 0.06% (*w*/*v*) sucrose fatty acid ester solution and degreased using ultrasound. After degreasing, they were rinsed repeatedly with pure water, and impurity proteins were removed in a 10% (*w*/*v*) NaCl solution. The purified pork skin was washed and drained, and then placed in a pressure cooker and simmered until softened. Subsequently, it was ground in a colloid mill to obtain unhydrolyzed collagen sol (CNS). A portion of CNS was taken, and a 400 U/mL papain solution was added. The mixture was hydrolyzed for 40 min in a constant-temperature water bath at 60 °C, and then heated at 100 °C for 10 min to inactivate the enzyme, yielding hydrolyzed collagen sol (CHS). Another portion of CHS was taken, and a 20 U/mL transglutaminase (TGase) solution was added. The mixture was cross-linked at 45 °C for 2 h. After cross-linking, the enzyme was inactivated in a boiling water bath to obtain a hydrolyzed cross-linked collagen gel (CHG) with a final TGase concentration of 0.5 wt%.

### 2.3. Design of Animal Experiments

Male C57BL/6J mice (8 weeks old, weighing 23 ± 1 g) were randomly divided into four groups (control group (Con), CNS, CHS, and CHG), with 25 mice in each group, and acclimated for one week under standard housing conditions (temperature 22 ± 2 °C, relative humidity 55% ± 5%, and a 12-h light/dark cycle). During the acclimation period, the mice had free access to sufficient food and drinking water. Prior to the start of the formal experiment, the mice were fasted for 12 h but allowed free access to water. Animals in the experimental groups were gavaged with a dose of 1 g/kg (dry weight); the Con group underwent gavage without receiving any sample. Blood samples were collected at 0 min, 30 min, 90 min, 3 h, and 6 h after gastric intubation, and the mice were euthanized and dissected (*n* = 5 at each time point). The 0-h samples were collected from mice in the blank control group that did not receive collagen. All blood samples were collected as terminal samples, with each mouse providing a sample at only one time point. Serum was isolated from the collected blood samples for the determination of serum peptide levels and biochemical analysis (gastrointestinal hormones and neurotransmitters). Concurrently, gastric tissue was dissected from the mice, and digestive contents from the gastrointestinal tract were collected and stored at –80 °C. Gastric antrum tissue was obtained for RT-qPCR experiments. The average particle size of gastric contents was determined using the method described by Leng et al. [11]. Dilute the supernatant of the gastric contents with saline to a 1:10 (*v*/*v*) ratio, transfer it to a cuvette, and analyze it using a Malvern laser particle size analyzer (Nano ZS90, Malvern Instruments, UK). The protein concentration in the chyle supernatant was determined using a bicinchoninic acid assay (BCA) kit (Jiancheng, Nanjing, China).

### 2.4. In Vivo Digestion Assay of Collagen Gel

Forty-eight male C57BL/6J mice were randomly divided into four groups of 12 mice each for in vivo experiments. Mice in the control group (Con) were gavaged with saline containing 50 μM Rhodamine B, while mice in the CNS, CHS, and CHG groups were gavaged with CNS, CHS, and CHG samples, respectively, uniformly mixed with 50 μM Rhodamine B. Similarly, after oral administration, the mice were dissected at 30 min, 90 min, and 180 min following the procedure to obtain gastrointestinal tissues. Using an in vivo imaging system, the fluorescence signal intensity (ROI) values of gastric tissue in each group were recorded. The gastrointestinal transit rate was calculated using the following formula: Gastrointestinal transit rate = (Length of the fluorescent digestive tract/Total length of the digestive tract) × 100%.

### 2.5. Assay of Gastrointestinal Hormones and Neurotransmitters

Determine the levels of gastrointestinal hormones (leptin (LEP) and cholecystokinin (CCK)) and neurotransmitters (vasoactive intestinal peptide (VIP) and serotonin (5-HT)) in mouse serum using the method provided by the enzyme-linked immunosorbent assay (ELISA) kit.

### 2.6. Gene Detection in the Gastric Antrum of Mice

Tissue samples from the gastric antrum were collected immediately after execution, rapidly frozen in liquid nitrogen, and stored at −80 °C until analysis. Total RNA was extracted from 50 mg of gastric antrum tissue using the RNA Easy Fast Kit (Tiangen, DP451) according to the manufacturer’s instructions. RNA quality was assessed using a micro-spectrophotometer (CFX 384 Touch, Bio-Rad, USA); the A260/A280 ratio for all samples ranged from 1.8 to 2.0. Subsequently, cDNA was synthesized via reverse transcription using the All-In-One 5X RT MasterMix Kit (abm, catalog no. G592) and stored at −20 °C. After cDNA synthesis, RT-qPCR was used to detect the expression levels of genes such as hyperpolarization-activated cyclic nucleotide-gated cation channel 1 (HCN1), transient receptor potential M7 (Trpm7), gap junction protein 43 (CX43), and receptor tyrosine kinase (c-Kit) in the gastric antrum. RT-qPCR was performed on a PCR instrument (P100+, Pultton, USA) using the Real-Time Fluorescent Quantitative PCR Kit (Novoprotein, E096-01A). The amplification efficiency of all primers ranged from 90% to 110%, with a correlation coefficient (r^2^) > 0.99. Primer specificity was confirmed via melting curve analysis, with each primer pair exhibiting a single peak. Relative expression levels were calculated using the 2^−ΔΔCt^ method. The number of biological replicates per time point was n = 5. The specific primer sequences are listed in the table below.

### 2.7. Immunohistochemical Analysis

Immunohistochemical analysis was performed on the c-kit protein. Paraffin-embedded gastric antrum tissue sections were sequentially deparaffinized with graded ethanol solutions, followed by rinsing with distilled water to remove the ethanol. Antigen retrieval was performed on the sections. After cooling, the slides were placed in PBS (pH 7.4) and washed in a shaking bath. Circles were then drawn on the slides using an immunohistochemistry pen (G6100). Hydrogen peroxide solution was added to the circles on the sections, and the sections were incubated at room temperature in the dark for 15 min to block endogenous peroxidase. Normal goat serum was added to the circles for blocking at room temperature. A negative control was prepared by substituting PBS for the primary antibody. The blocking solution was gently tapped off, the primary antibody (Servicebio, GB153799, 1:500) was added, and the sections were incubated overnight at 4 °C. After excess liquid was lightly tapped off, the secondary antibody (Servicebio, G1302, ready-to-use) was added to the circles to cover the tissue, and the sections were incubated in an oven. After excess liquid was lightly tapped off, DAB color development solution was added to the circles, and the sections were rinsed to stop the color reaction. After excess solution was gently tapped off, DAB staining solution was added to the well, and the slides were rinsed to stop the reaction. Finally, the slides were mounted with neutral resin. The results were examined under a white-light microscope (SWE-CX63), and the slides were stored. High-magnification fields were randomly selected from each section by two independent observers who were unaware of the experimental group status, and the fields were evaluated. The H-score was calculated as: H-score = Σ (pi × i), where i represents the staining intensity (0, 1, 2, or 3), and pi represents the percentage of cells with that intensity.

### 2.8. Serum Collagen Dipeptide/Tripeptide Assay

Transfer 100 µL of plasma into a centrifuge tube, add 300 µL of anhydrous ethanol, centrifuge at 3000× *g* for 10 min, aspirate the supernatant, filter it through a 0.22 µm organic membrane, and transfer it to a brown sample vial as the sample to be analyzed. A liquid chromatography–triple quadrupole tandem mass spectrometer (Waters Xevo TQ) was used. An in-line filter (2.1 mm, 0.2 µm) was placed upstream of the column, which was an ACQUITY UPLC HSS T3 column (1.8 µm, 2.1 mm × 150 mm) set to a column temperature of 40 °C. The mobile phase flow rate was set to 0.35 mL/min, and the injection volume was 5 µL. The mobile phase consisted of Phase A (aqueous solution containing 0.05% formic acid) and Phase B (95% acetonitrile solution containing 0.1% formic acid). The elution program was as follows (Table 2):

Mass spectrometry analysis was performed using an electrospray ionization (ESI) source in positive ion mode. Sheath gas flow rate: 10 L/min (temperature: 325 °C). Nebulizer pressure: 241 kPa. Dry gas flow rate: 8 L/min (temperature: 300 °C). Capillary voltage: 4000 V. The MRM parameter settings for the selected dipeptide and tripeptide standards are shown in Table 3. A series of concentration solutions was prepared using six target peptide standards (purity ≥ 98%), and a good linear relationship was observed over a wide concentration range, with correlation coefficients all greater than 0.99. The limits of detection (LODs) and limits of quantification (LOQs) were determined based on signal-to-noise ratios (S/N) of ≥3 and ≥10, respectively; the LOD and LOQ for both dipeptides and tripeptides met the requirements for quantitative analysis. Spiked recovery tests at low, medium, and high concentration levels showed that the recovery rates for proline-containing dipeptides and hydroxyproline-containing dipeptides were both within acceptable ranges, with low relative standard deviations (RSDs). Intra-day and inter-day precision, expressed as RSD, were both below the acceptable standards for bioanalytical method validation. 

### 2.9. Antioxidant Evaluation of Collagen Dipeptides and Tripeptides

Using the maximum concentration of collagen dipeptides and tripeptides in CHG serum as the reference ratio, we prepared Mixture A (×1), Mixture B (×50, with each monopeptide concentration set at 50 times the maximum concentration), Gly-Pro (×50), Hyp-Gly (×50), Pro-Hyp (×50), Glu-Hyp (×50-fold), Ser-Hyp-Gly (×50-fold), and Gly-Pro-Hyp (×50-fold). Mixture A (×1) was formulated based on the maximum serum concentrations of the six peptides measured in the CHG group and was designed to simulate physiological exposure conditions. The concentration of Mixture B (×50) was set at 50 times that of Mixture A to assess whether these peptides exhibit measurable antioxidant activity and concentration-dependent effects in vitro. The ×50 concentration is not intended to represent physiological levels but is used solely for in vitro mechanism exploration. The in vitro antioxidant capacity of each peptide solution was assessed using the Hydroxyl Radical Scavenging Assay Kit (Grace, G0125W) and the Total Antioxidant Capacity Assay Kit (Grace, G0142W).

Cell Antioxidant Assay: First, solutions of peptides A and B mixed in various dilutions were used to conduct biocompatibility tests between the peptide solutions and skin fibroblasts (HSF, Procell). Subsequently, skin fibroblasts were selected, and the experiment was organized into the following groups: control group: normal culture without H_2_O_2_ or peptides; model group: induced oxidative damage with H_2_O_2_ alone, without the mixed-peptide solution; mixed-peptide solution group A (×1): pretreatment with mixed-peptide solution A + H_2_O_2_ damage; mixed-peptide solution B group (×50): pretreatment with mixed-peptide solution B + H_2_O_2_ damage. Antioxidant capacity was evaluated using ROS immunofluorescence staining.

### 2.10. Statistics and Analysis

Statistical analysis was performed using Origin (version 2021; Origin Lab Corporation, USA), and data are presented as mean ± standard error. Differences among groups were analyzed using one-way analysis of variance (ANOVA), followed by Tukey’s post hoc test. A *p*-value of less than 0.05 was considered statistically significant.

## 3. Results

### 3.1. Gastric Retention and Intestinal Transit

**Figure 1:**
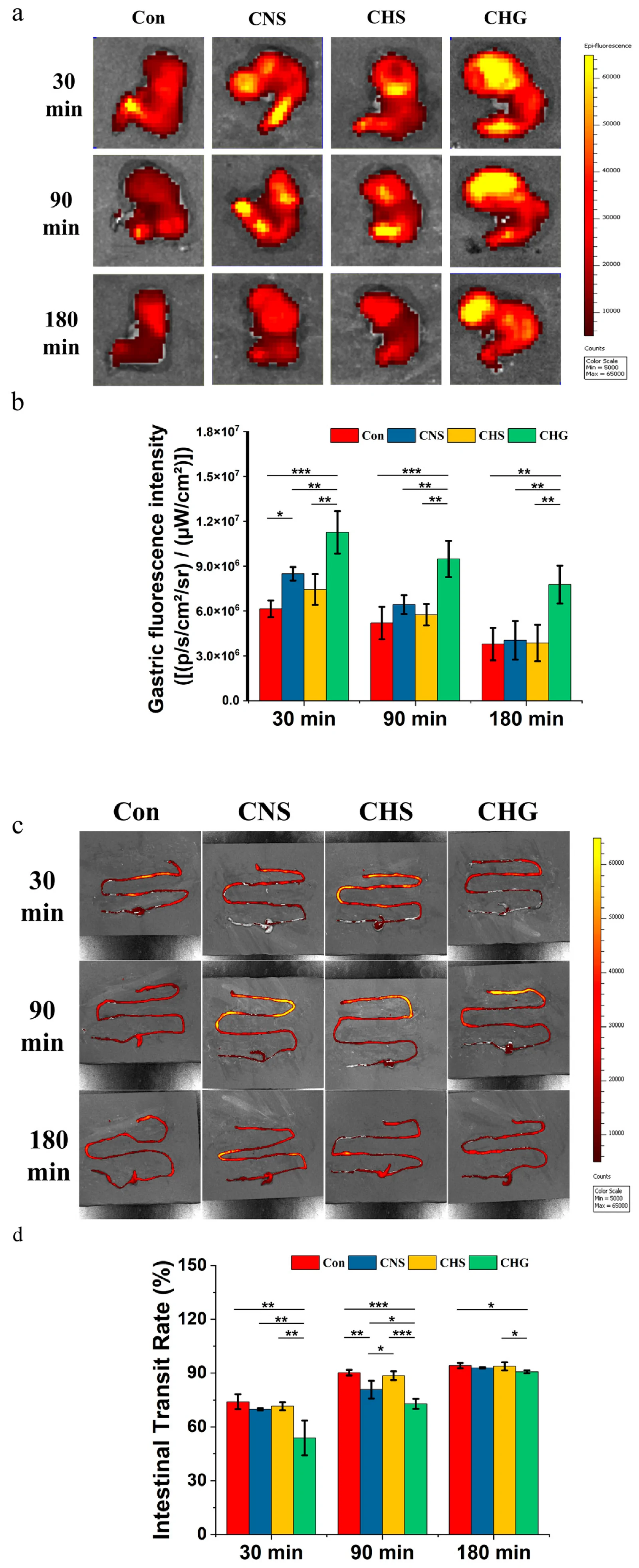
Analysis of gastrointestinal emptying behavior in vivo (*n* = 4). (**a**) Fluorescence imaging of gastric tissue for unhydrolyzed collagen sol (CNS), hydrolyzed collagen sol (CHS), and TGase-crosslinked collagen gel (CHG) at 30, 90, and 180 min; (**b**) fluorescence intensity in gastric tissue; (**c**) intestinal transit of CNS, CHS, and CHG after 30, 90, and 180 min of digestion; (**d**) intestinal transit rate. * *p* < 0.05, ** *p* < 0.01, and *** *p* < 0.001. Data are presented as mean ± standard error.

The intragastric fluorescence intensity was measured at 30, 90, and 180 min after oral administration to compare gastric retention among the Con, CNS, CHS, and CHG groups (Figure 1a,b). At 30 min, the CHS group showed no significant difference from the Con group, whereas the CNS group exhibited significantly higher fluorescence intensity than the Con group (*p* < 0.05). The CHG group displayed significantly higher intragastric fluorescence intensity than the CNS group (*p* < 0.01) and the CHS group (*p* < 0.01). At 90 min, fluorescence intensity in the Con group had decreased to a level similar to that observed at 180 min. The CNS and CHS groups also showed decreased intensity, with no significant difference from the Con group. In contrast, the CHG group remained significantly higher than the Con group (*p* < 0.001), the CNS group (*p* < 0.01), and the CHS group (*p* < 0.01). At 180 min, the fluorescence intensity in the CHG group had gradually declined but was still significantly higher than that in the CNS group (*p* < 0.01) and the CHS group (*p* < 0.01). Throughout the observation period, CHG exhibited extremely slow and sustained emptying kinetics.

Intestinal transit rates were calculated based on the distribution of fluorescent dye in the intestine (Figure 1c,d). At 30 min, the transit rates in the CNS and CHS groups were 69.84% and 71.55%, respectively, with no significant difference from the Con group. The CHG group showed a transit rate of 53.79%, significantly lower than the CNS group (*p* < 0.01) and the CHS group (*p* < 0.01). At 90 min, the Con and CHS groups reached transit rates of 90.21% and 88.55%, respectively, while the CNS group reached 80.79%. The CHG group showed a rate of 72.83%, significantly lower than the CNS group (*p* < 0.05) and the CHS group (*p* < 0.001). At 180 min, the transit rates of the Con, CHS, and CNS groups were 94.15%, 92.90%, and 93.70%, respectively. The CHG group reached 90.67%, which was still significantly lower than the CHS group (*p* < 0.05). Therefore, the cross-linked gel structure of CHG not only produces a significant prolonged gastric retention effect but also maintains a slower transit rate in the small intestine.

### 3.2. Analysis of the Physicochemical Properties of Gastric Contents

**Figure 2:**
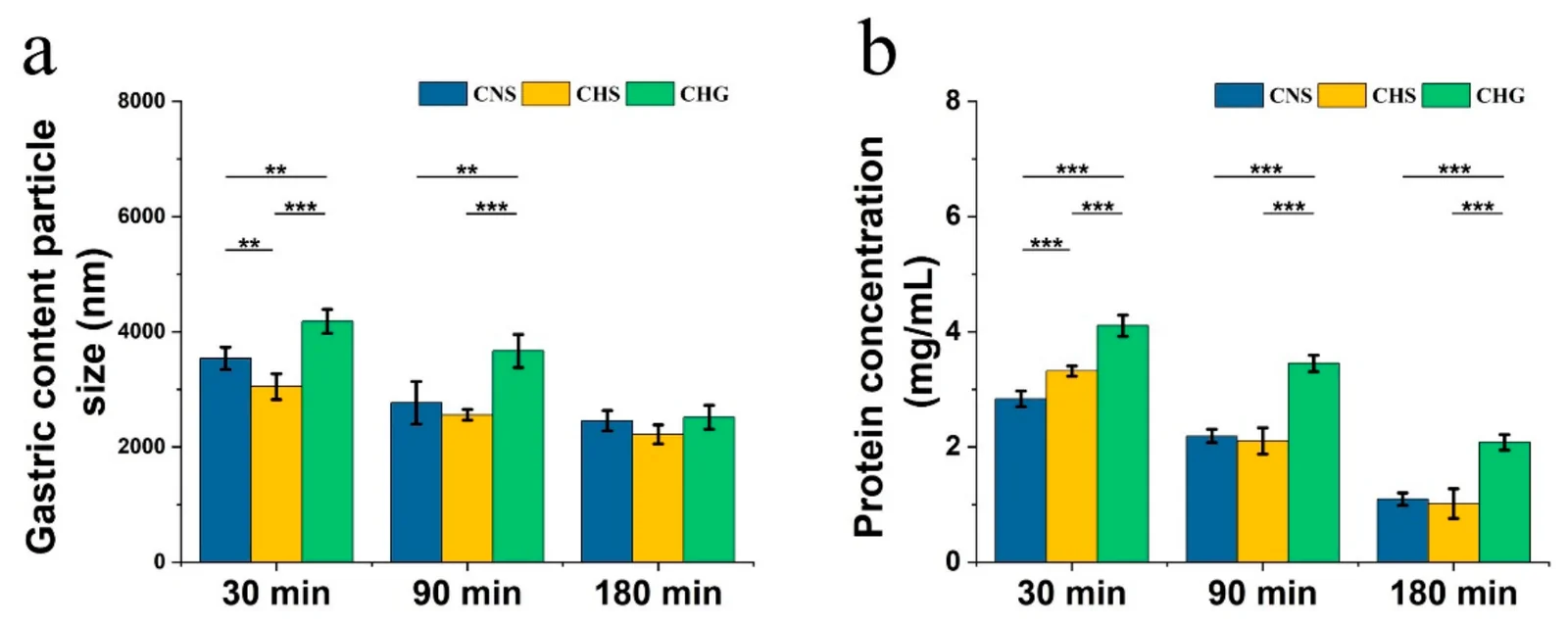
Physicochemical properties of gastric contents. (**a**) Particle size of gastric contents (*n* = 5); (**b**) protein concentration in the supernatant of gastric contents (*n* = 5). * *p* < 0.05, ** *p* < 0.01, and *** *p* < 0.001. Data are presented as mean ± standard error.

The mean particle size of gastric contents was measured at 30, 90, and 180 min after gavage (Figure 2a). At 30 min, the mean particle size in the CNS group was 3537.13 nm, significantly larger than that in the CHS group (3048.00 nm, *p* < 0.01). The CHG group showed a mean particle size of 4370.00 nm, significantly larger than both the CNS group (*p* < 0.01) and the CHS group (*p* < 0.001). At 90 min, the particle sizes in the CNS and CHS groups decreased to 2766.80 nm and 2556.53 nm, respectively, with no significant difference between them. The CHG group had a mean particle size of 3666.33 nm, significantly larger than the CNS group (*p* < 0.01) and the CHS group (*p* < 0.001). At 180 min, the CHG group still showed a mean particle size of 2513.87 nm, larger than that of the CHS group, but no difference from the CNS group. This trend in particle size indicates that the disintegration of CHG in the stomach is a continuous but slow process.

The protein concentration in the supernatant of gastric contents was also determined (Figure 2b). At 30 min, the protein concentrations in the CNS and CHS groups were 2.83 mg/mL and 3.32 mg/mL, respectively, with the CHS group significantly higher than the CNS group (*p* < 0.001). The CHG group had a protein concentration of 4.11 mg/mL, significantly higher than both the CNS group (*p* < 0.001) and the CHS group (*p* < 0.001). At 90 min, the CNS and CHS groups showed protein concentrations of 2.19 mg/mL and 2.10 mg/mL, respectively, with no significant difference. The CHG group (3.45 mg/mL) remained significantly higher than the CNS (*p* < 0.001) and CHS (*p* < 0.001) groups. At 180 min, the protein concentrations in the CNS, CHS, and CHG groups were 1.09 mg/mL, 1.02 mg/mL, and 2.08 mg/mL, respectively, with the CHG group significantly higher than the CNS (*p* < 0.001) and CHS (*p* < 0.001) groups. Clearly, CHG remains in the stomach for a longer period of time, giving pepsin more time to continuously act on the gel network, gradually converting insoluble collagen into soluble peptides and releasing them into the supernatant.

### 3.3. Analysis of Gene and Protein Expression Associated with the Gastric Antrum

**Figure 3:**
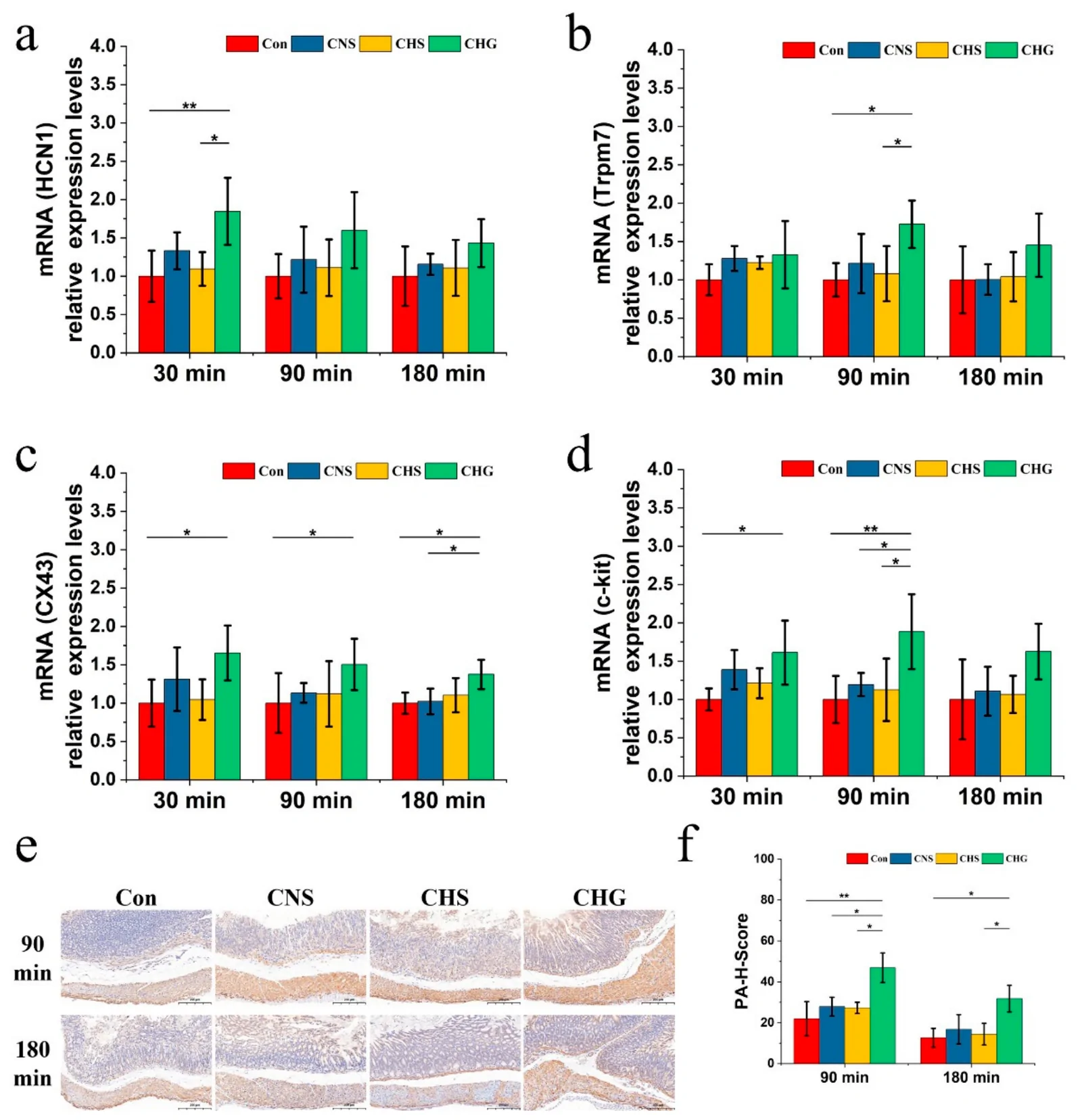
Analysis of gene and protein expression associated with Cajal interstitial cells (ICCs) in the gastric antrum. (**a**) Relative expression levels of hyperpolarization-activated cyclic nucleotide-gated cation channel 1 (HCN1) mRNA (*n* = 5); (**b**) relative expression levels of transient receptor potential melastatin 7 (Trpm7) mRNA (*n* = 5); (**c**) relative expression levels of connexin 43 (CX43) mRNA (*n* = 5); (**d**) relative expression levels of receptor tyrosine kinase (c-kit) mRNA (*n* = 5); (**e**) representative images of c-kit immunohistochemistry (*n* = 3); (**f**) PA-H-Score values for c-kit immunohistochemistry (*n* = 3). * *p* < 0.05, ** *p* < 0.01, and *** *p* < 0.001. Data are presented as mean ± standard error.

The mRNA expression levels of HCN1, Trpm7, CX43, and c-kit in gastric antral tissue were measured by RT-qPCR (Figure 3a–d). For HCN1, the CHG group showed higher expression than the Con group at 30 min (*p* < 0.01), the CNS group, and the CHS group (*p* < 0.05). At 90 and 180 min, HCN1 expression in the CHG group remained higher than the Con, CNS and CHS groups but not significantly different. For Trpm7, no significant differences were observed among groups at 30 or 180 min. At 90 min, the CHG group showed significantly higher Trpm7 expression than the Con group (*p* < 0.05) and the CHS group (*p* < 0.05). For CX43, the CHG group exhibited significantly higher expression than the Con group at 30 and 90 min (*p* < 0.05). At 180 min, CX43 expression in the CHG group was significantly higher than in the Con group (*p* < 0.05) and the CNS group (*p* < 0.05). For c-kit, the CHG group showed significantly higher expression than the Con group at 30 min (*p* < 0.05). At 90 min, c-kit expression in the CHG group was significantly higher than in the Con (*p* < 0.01), CNS (*p* < 0.05), and CHS (*p* < 0.05) groups; no significant differences were observed among groups at 180 min.

Immunohistochemical analysis of c-kit protein was performed on gastric antral tissue sections (Figure 3e). The H-score (Figure 3f) for c-kit in the CHG group at 90 min was significantly higher than that in the Con group (*p* < 0.01), the CNS group (*p* < 0.05), and the CHS group (*p* < 0.05); that in the CHG group at 180 min was significantly higher than that in the Con group (*p* < 0.05) and the CHS group (*p* < 0.05). These data indicate that CHG treatment is associated with changes in the expression of ICC-related markers.

### 3.4. Analysis of Gastrointestinal Hormones and Neurotransmitters

**Figure 4:**
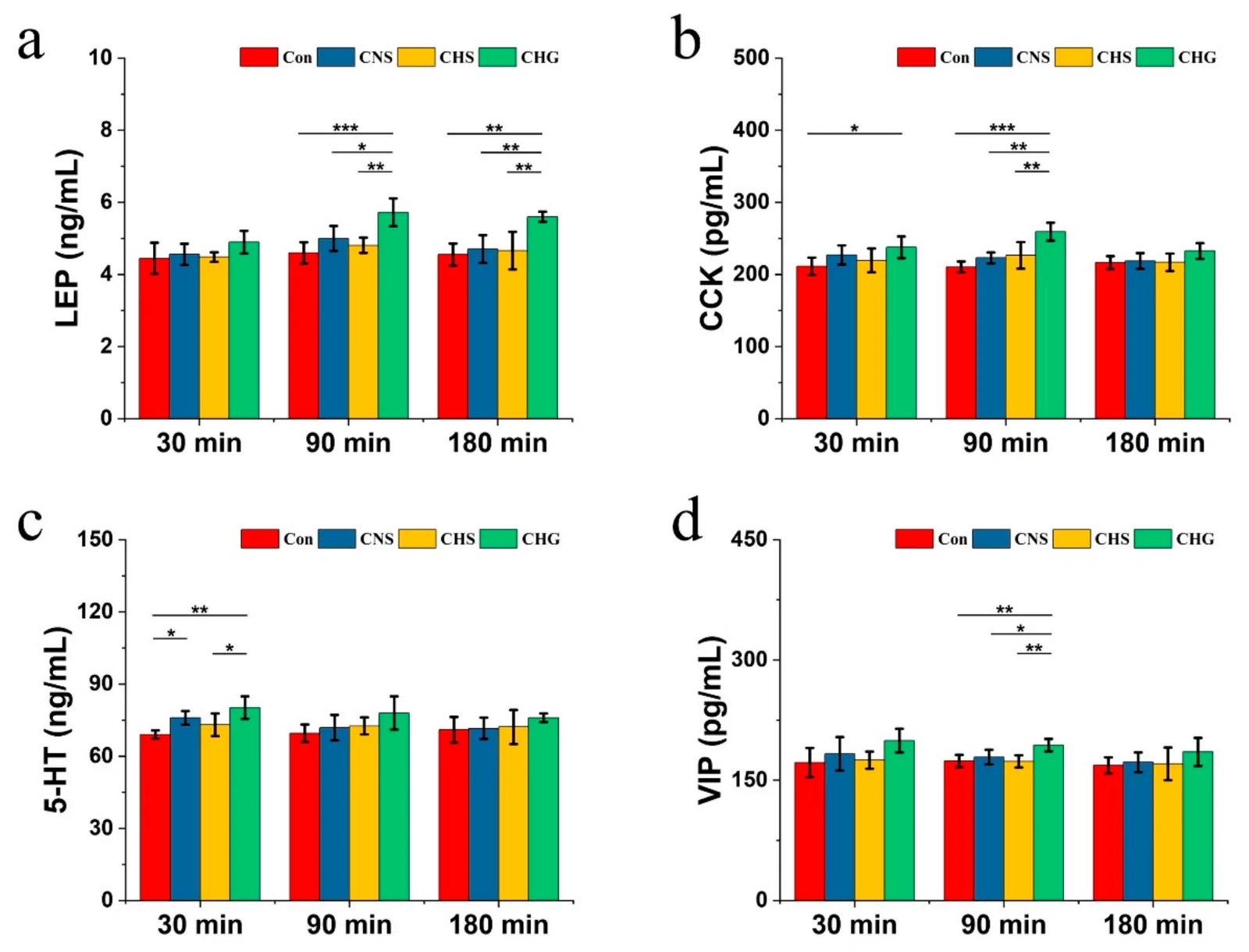
Effects of CHG on the secretion of gastrointestinal hormones (*n* = 5): (**a**) serum leptin (LEP) concentration; (**b**) serum cholecystokinin (CCK) concentration. Effects of CHG on neurotransmitters related to gastric retention: (**c**) serum serotonin (5-HT) concentration; (**d**) serum vasoactive intestinal peptide (VIP) concentration. * *p* < 0.05, ** *p* < 0.01, and *** *p* < 0.001. Data are presented as mean ± standard error.

Serum concentrations of LEP, CCK, 5-HT, and VIP were measured by ELISA (Figure 4a–d). At 30 min, the CHG group had higher serum LEP than the Con, CNS and CHS groups, but it was not significantly different. At 90 min, LEP levels in the CHG group were significantly higher than those in the Con group (*p* < 0.001), the CNS group (*p* < 0.05), and the CHS group (*p* < 0.01). At 180 min, LEP levels in the CHG group were significantly higher than those in the Con group (*p* < 0.01), the CNS group (*p* < 0.01), and the CHS group (*p* < 0.01). The CNS and CHS groups did not differ significantly from the Con group at any time point. For CCK, the CHG group showed significantly higher levels than the Con group at 30 min (*p* < 0.05). At 90 min, CCK in the CHG group peaked and was significantly higher than in the Con group (*p* < 0.001), CNS group (*p* < 0.01), and CHS group (*p* < 0.01). At 180 min, the CHG group remained higher than the Con and CHS groups. The CNS and CHS groups did not differ significantly from the Con group.

Serum 5-HT in the CHG group was significantly higher than in the Con group (*p* < 0.01) and the CHS group (*p* < 0.05) at 30 min. The CNS group also showed significantly higher 5-HT than the Con group at this time point (*p* < 0.05). At 90 min, the CHG group remained higher than the Con group, while the CNS and CHS groups returned to baseline. At 180 min, only the CHG group maintained a higher level. The VIP levels in the CHG group were higher than in the Con group and the CHS group at 30 min. At 90 min, the VIP in the CHG group was significantly higher than in the Con group (*p* < 0.01), the CNS group (*p* < 0.05), and the CHS group (*p* < 0.01). At 180 min, the CHG group remained slightly higher than the Con group.

Overall, the temporal patterns of gastrointestinal hormone and neurotransmitter secretion in the CHG group were consistent with the slow-progression kinetics observed in the gastrointestinal tract.

### 3.5. Serum Appearance of Collagen Di- and Tripeptides

**Figure 5:**
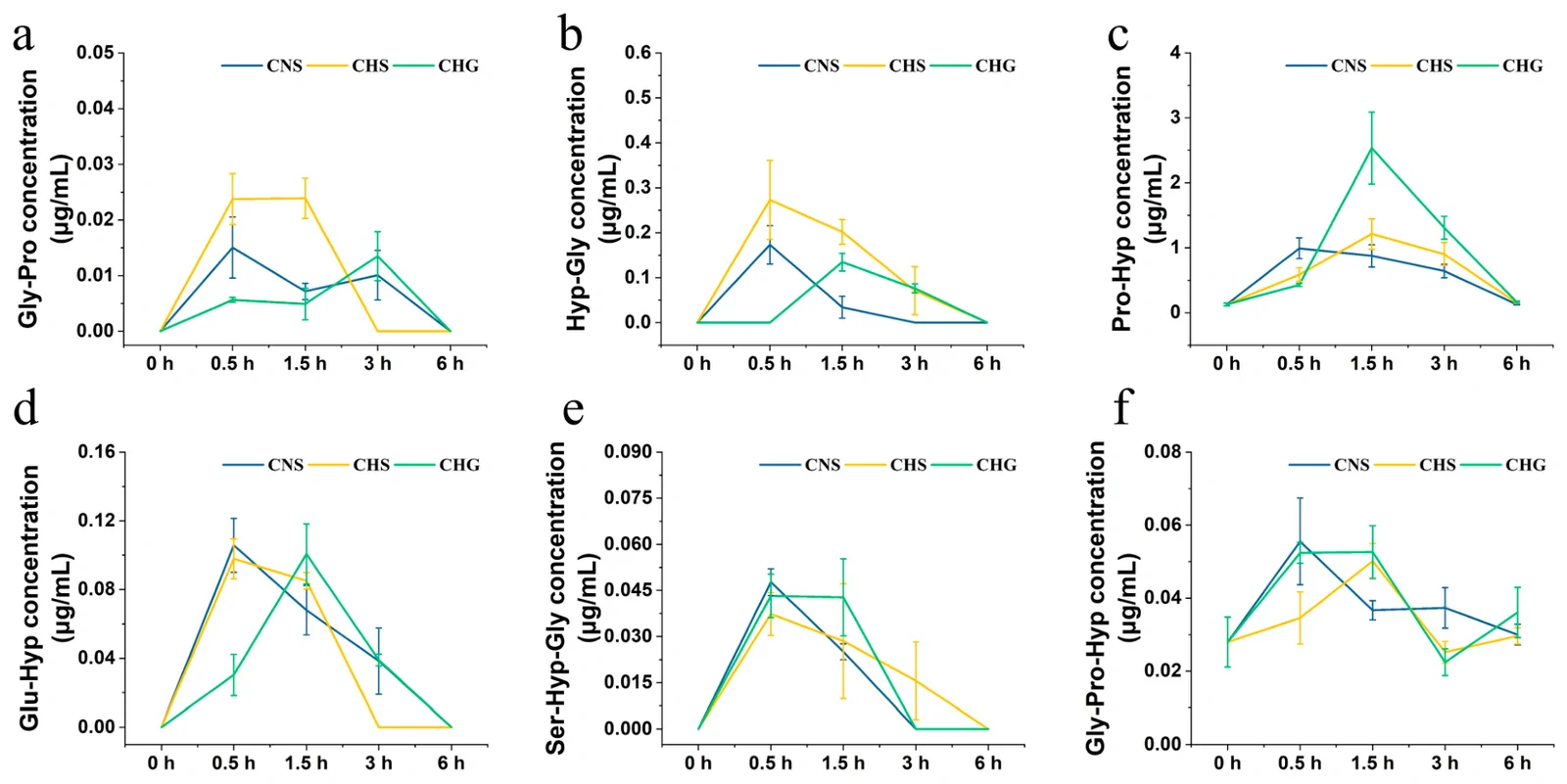
CHG breaks down collagen dipeptides and tripeptides in serum at different time points (*n* = 5). (**a**) Serum Gly-Pro concentration; (**b**) serum Hyp-Gly concentration; (**c**) serum Pro-Hyp concentration; (**d**) serum Glu-Hyp concentration; (**e**) serum Ser-Hyp-Gly concentration; (**f**) serum Gly-Pro-Hyp concentration. Data are presented as mean ± standard error.

Serum samples were collected from individual mice at 0, 0.5, 1.5, 3, and 6 h after gavage (*n* = 5 at each time point) for peptide quantification. Serum concentrations of Gly-Pro, Hyp-Gly, Pro-Hyp, Glu-Hyp, Ser-Hyp-Gly, and Gly-Pro-Hyp were quantified at 0, 0.5, 1.5, 3, and 6 h after administration (Figure 5a–f). Among the four dipeptides, Pro-Hyp showed the highest serum concentration at all time points. In the CHG group, Pro-Hyp reached 0.59 µg/mL at 0.5 h, peaked at 2.54 µg/mL at 1.5 h, remained at 1.31 µg/mL at 3 h, and declined to 0.16 µg/mL at 6 h. The peak concentration in the CNS and CHS groups was 0.99 µg/mL and 1.21 µg/mL, respectively, both lower than that in the CHG group. For Hyp-Gly, Gly-Pro, and Glu-Hyp, the CNS and CHS groups reached maximum concentrations at 0.5 h, followed by a gradual decline. In the CHG group, Hyp-Gly and Glu-Hyp peaked at 1.5 h, while Gly-Pro peaked at 3 h. Among the tripeptides, serum Ser-Hyp-Gly peaked at 0.5 h in all three groups. The CNS and CHS groups declined rapidly by 1.5 h, whereas the CHG group maintained relatively high levels. For Gly-Pro-Hyp, baseline levels of approximately 0.03 µg/mL were detected in all three groups before gavage. In the CHG group, Gly-Pro-Hyp rose to 0.05 µg/mL at 0.5 h and 1.5 h, and then slowly declined to 0.04 µg/mL at 6 h. These results indicate that CHG digestion led to a more sustained serum appearance of collagen di- and tripeptides.

### 3.6. Analysis of the Antioxidant Properties of Absorbed Collagen Dipeptides and Tripeptides

**Figure 6:**
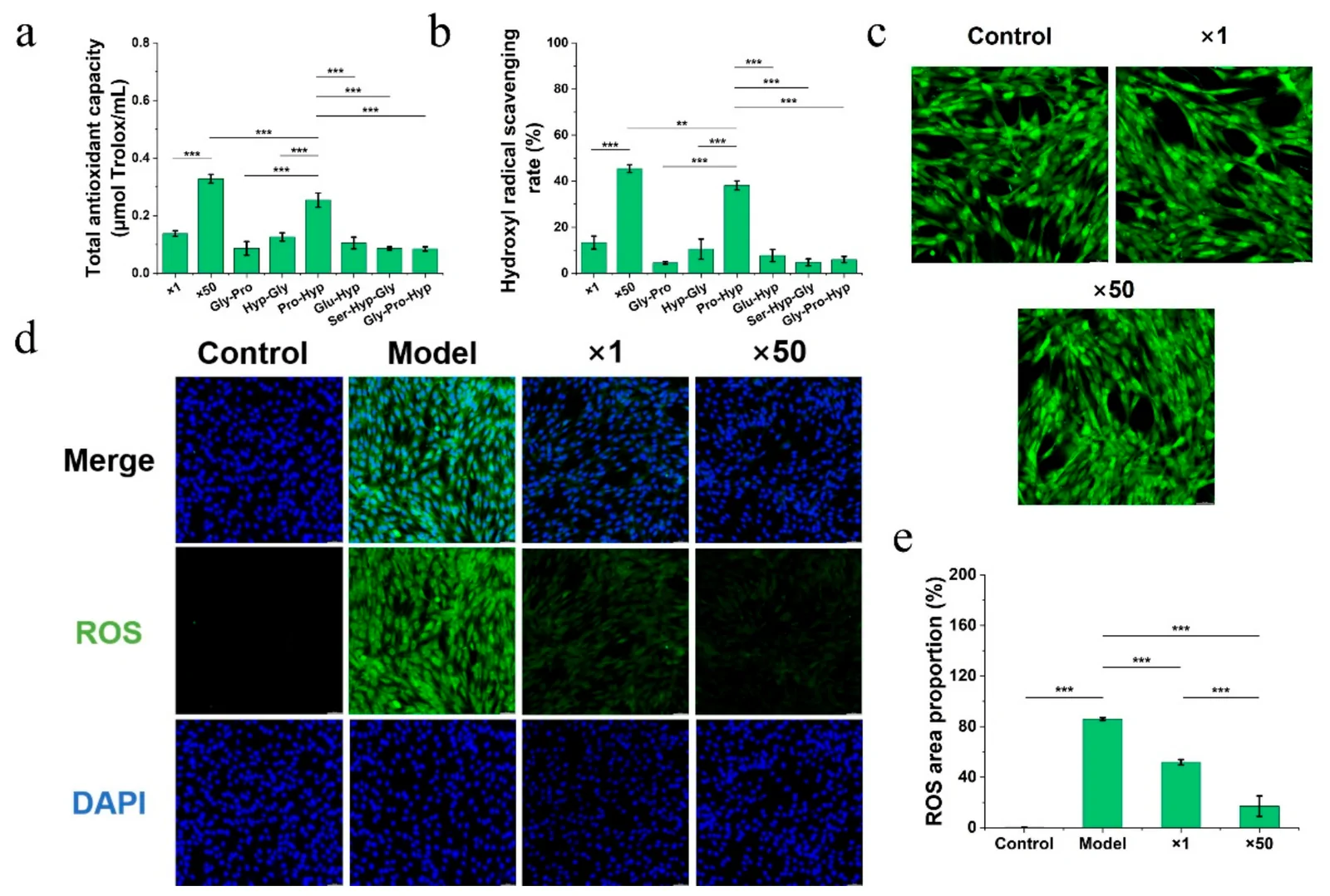
In vitro evaluation of antioxidant effects (*n* = 5). (**a**) Total antioxidant capacity of collagen dipeptides and tripeptides; (**b**) ability of collagen dipeptides and tripeptides to scavenge hydroxyl radicals; (**c**) cytocompatibility of collagen dipeptide and tripeptide systems with dermal fibroblasts; (**d**,**e**) evaluation of the antioxidant activity of collagen dipeptide and tripeptide systems on dermal fibroblasts. * *p* < 0.05, ** *p* < 0.01, and *** *p* < 0.001. Data are presented as mean ± standard error.

The total antioxidant capacity and hydroxyl radical scavenging activity of individual collagen di-/tripeptides and their mixtures were evaluated (Figure 6a,b). Mixed-peptide solution B (×50) exhibited a total antioxidant capacity of 0.58 µmol Trolox/mL, significantly higher than mixed-peptide solution A (×1, 0.14 µmol Trolox/mL). Among the single peptides, Pro-Hyp had the highest total antioxidant capacity (0.46 µmol Trolox/mL), followed by Glu-Hyp (0.20 µmol Trolox/mL) and Hyp-Gly (0.24 µmol Trolox/mL). Gly-Pro, Ser-Hyp-Gly, and Gly-Pro-Hyp each had a total antioxidant capacity of 0.17 µmol Trolox/mL. For hydroxyl radical scavenging, mixed-peptide solution B showed the highest scavenging rate (45.46%), significantly higher than mixed-peptide solution A (13.29%). Among the single peptides, Pro-Hyp exhibited the highest scavenging rate (38.17%), followed by Hyp-Gly (10.52%). The scavenging rates of the other peptides were below 8% (Gly-Pro 4.56%; Glu-Hyp 7.73%; Ser-Hyp-Gly 4.77%; Gly-Pro-Hyp 5.98%).

Cell biocompatibility experiments showed that neither mixed-peptide solution A nor B inhibited the viability of skin fibroblasts within the tested concentration range (Figure 6c). In the H_2_O_2_-induced oxidative damage model, the ROS fluorescence area in the model group was significantly higher than that in the control group (*p* < 0.001). Pretreatment with mixed-peptide solution A significantly reduced the ROS fluorescence area (*p* < 0.001), and mixed-peptide solution B showed an even greater reduction, indicating a concentration-dependent effect (Figure 6d,e). These findings indicate that the CHG-enriched peptide mixture exhibited antioxidant capacity in in vitro chemical and cellular assays.

## 4. Discussion

The present study first demonstrated that, compared with CNS and CHS, CHG exhibited greater gastric retention and slower intestinal transit, accompanied by larger gastric content particles and more sustained protein presence in the stomach. However, it should be noted that the Rhodamine B dye is mixed with the sample rather than covalently bound to it. The strong noncovalent interactions between Rhodamine B and collagen support the notion that it partially binds to the gel network during digestion. Nevertheless, the possibility of partial dye diffusion cannot be ruled out. Nevertheless, the consistent differences in fluorescence retention rates and intestinal transit times observed among the Con, CNS, CHS, and CHG groups suggest that the behavior of the tracer is largely influenced by the physical structure of the collagen matrix, rather than solely reflecting the diffusion of free dye. These findings suggest that the cross-linked gel structure of CHG fundamentally alters the physical processing of collagen in the gastrointestinal tract. The three-dimensional network structure formed by covalent cross-linking gives CHG extremely high mechanical stability, enabling it to resist fragmentation caused by gastric peristalsis and the penetration of gastric juice during the early stages of digestion [12,13]. In contrast, uncrosslinked CNS and prehydrolyzed CHS empty more quickly due to their lower mechanical strength and smaller initial particle size. Physicochemical analysis of the gastric contents further supported this explanation: at 30 and 90 min, the average particle size in the CHG group was significantly larger than that in the CNS and CHS groups, indicating a slower disintegration rate. Even after 180 min, the CHG-derived particles remained larger than the CHS-derived particles. This slow degradation process is consistent with the view that gastric retention of protein gels depends on a reduction in particle size; only particles small enough to pass through the pylorus can be emptied [13]. Protein concentrations in the gastric supernatant of the CHG group remained consistently high, indicating that pepsin was continuously hydrolyzing the gel network and gradually releasing soluble peptides. This pattern of slow dissociation and continuous hydrolysis is consistent with previously reported findings, namely that the swelling of protein gels enhances the diffusion of acid and pepsin, thereby improving digestive efficiency [14,15,16,17]. In addition to delaying gastric retention, CHG also slowed intestinal motility. At 30 and 90 min, the intestinal transit rate in the CHG group was significantly lower than that in the CNS and CHS groups, and this difference remained statistically significant at 180 min. This longer intestinal transit time likely provides a longer window for enzymatic degradation. Lu et al. also observed that cross-linked collagen gels were continuously degraded by intestinal proteases as they passed through the gut, and characteristic peptide fragments could still be detected in the cecum [7].

The CHG causes slowed gastric retention, accompanied by changes in the expression of genes associated with ICCs in the gastric antrum as well as in serum hormone and neurotransmitter levels, suggesting that signals related to gastrointestinal motility may be involved in the retention effect of CHG. In the CHG group, mRNA levels of HCN1, Trpm7, CX43, and c-kit were significantly upregulated at multiple time points, and c-kit protein levels were also elevated. ICCs are pacemaker cells in the gastrointestinal tract, and the coordinated expression of their ion channels and signaling proteins is essential for generating the slow-wave potentials that drive rhythmic gastric contractions [8,10,18,19,20,21]. The results of this study are consistent with the possibility that the persistent presence of CHG in the stomach exerts a long-lasting mechanical stimulus on the ICCs, which may be associated with enhanced pacemaking activity and increased electrical coupling. In contrast, no similar upregulation was observed in the CNS and CHS groups that underwent rapid gastric retention. This finding is consistent with previous evidence that food texture and gel strength can modulate gastric antral motility and its associated gene expression [11]. The characteristics of serum levels of LEP, CCK, 5-HT, and VIP also support the involvement of the brain–gut axis. CHG caused sustained elevations in the levels of these hormones and neurotransmitters, whereas the CNS and CHS groups exhibited only transient changes or no significant differences compared with the Con group. LEP is secreted by gastric glands and is known to be released in response to gastric distension and nutritional stimuli [22,23]. CCK is released from enteroendocrine cells in response to peptide stimuli and acts to delay gastric retention, allowing more complete digestion [24,25]. The continuous release of CCK in the CHG group may be related to the prolonged presence of peptides generated from the gel. 5-HT and VIP are also important regulators of gastrointestinal motility; 5-HT stimulates motility, while VIP mediates gastric accommodation and smooth muscle relaxation [26,27]. The sustained elevation of these markers in the CHG group coincided temporally with the observed delays in gastric retention and prolonged intestinal transit time. However, these data are merely correlational, and further intervention studies are needed to determine whether these markers are causal factors leading to changes in motility function. It should be emphasized that the present data provide correlational evidence; further pharmacological or genetic intervention studies would be required to establish the exact regulatory pathways.

A key finding of this study is that prolonged retention time of gastrointestinal contents is associated with the persistent presence of collagen-derived dipeptides and tripeptides in serum. Targeted LC-MS/MS analysis revealed distinct serum peptides among the three collagen preparations. In the CHG group, most collagen di- and tripeptides peaked between 0.5 and 1.5 h and then declined more gradually than in the CNS and CHS groups. Pro-Hyp, the major collagen peptide found in the bloodstream, showed the highest and most sustained serum concentration in the CHG group. At 3 h, the Pro-Hyp concentration in the CHG group remained higher than in the CNS and CHS groups. This prolonged serum appearance pattern is highly consistent with the prolonged gastric retention and slower intestinal transit observed in the CHG group. However, this parallel observation does not establish a causal relationship. It is possible that the cross-linked gel structure itself contributes to the persistent presence of these peptides by providing a continuous supply of substrates to proteases, a process that is independent of prolonged gastrointestinal motility. Only after undergoing thorough hydrolysis in the stomach can collagen be broken down into small peptide fragments that can be recognized by brush border enzymes and the PEPT1 transporter in intestinal epithelial cells [28]. In addition, TGase cross-linking alters the local conformation near the cross-linking sites (such as Lys644, Lys738, and Gln972), thereby exposing more proteolytic cleavage sites, which also promotes the release of absorbable collagen peptides in CHG [7,29]. The results of this study are consistent with previous pharmacokinetic studies, which show that Pro-Hyp has higher oral bioavailability than other collagen peptides [30]. Serum peptide levels were relatively low in the CNS group, most likely due to the low hydrolysis efficiency of intact collagen in the stomach, whereas the serum appearance observed in the CHS group reflects its prehydrolyzed nature and faster gastric retention rate.

Collagen peptides showed persistent presence, and they retained their antioxidant activity. In vitro antioxidant assays showed that, among the six peptides tested, Pro-Hyp—a serum peptide with the highest concentration and rich in CHG—exhibited the strongest total antioxidant capacity and hydroxyl radical scavenging activity. Solution B of the mixed-peptide mixture contained higher concentrations of the six characteristic peptides, and its antioxidant activity was significantly higher than that of Solution A, suggesting that its effect may be related to concentration rather than a clear synergistic effect. This observation suggests that the antioxidant capacity of this peptide mixture depends primarily on the concentration of Pro-Hyp, while the contributions of the other peptides are relatively minor. A cellular antioxidant model using H_2_O_2_ to induce oxidative damage in skin fibroblasts further confirmed the biological relevance of these peptides. Following pretreatment with a mixture rich in CHG peptides, the accumulation of reactive oxygen species (ROS) within the cells was significantly reduced in a concentration-dependent manner. These findings suggest that collagen dipeptides and tripeptides in serum following CHG intake may help protect skin cells from damage caused by oxidative stress [31,32]. However, since the peptide concentrations used in the cellular experiments were higher than physiological serum levels and no in vivo oxidative stress model was employed, these findings should be regarded as preliminary results and cannot be directly interpreted as indicating a skin-protective effect in vivo. This protective effect may be attributed to Pro-Hyp’s direct ability to scavenge free radicals, or to its modulatory role in intracellular antioxidant defense systems, although the latter was not directly measured in this study.

## 5. Conclusions

This study indicates that the CHG altered gastrointestinal retention and was associated with the persistent presence of certain collagen-derived dipeptides and tripeptides in mouse serum. Compared with unhydrolyzed and hydrolyzed collagen sols, CHG exhibited greater gastric retention, slower intestinal transit, larger gastric-content particles, and more sustained protein presence in the gastrointestinal tract. These effects occur concurrently with changes in ICC-related biomarkers and brain–gut axis signaling, although their causal contribution to altered gastrointestinal motility requires further investigation. Importantly, CHG is associated with the persistent presence of collagen dipeptides and tripeptides in serum, with Pro-Hyp showing the most prominent and persistent profile. The CHG-characteristic peptide mixture and individual peptides, particularly Pro-Hyp, also exhibited antioxidant activity in chemical assays and reduced H_2_O_2_-induced ROS accumulation in skin fibroblasts. Collectively, these findings suggest that a crosslinked collagen gel matrix can modulate gastrointestinal processing and prolong the systemic appearance of bioactive collagen peptides. These findings provide preliminary evidence for further exploration of CHG as a potential sustained-release food ingredient. 

## Figures and Tables

**Table 1:** Primer Sequences for Relevant Genes in the Gastric Antrum.

Primer Name	Forward Primer Sequence	Reverse Primer Sequence
β-actin	GGCTGTATTCCCCTCCATCG	CCAGTTGGTAACAATGCCATGT
HCN1	CAAATTCTCCCTCCGCATGTT	TGAAGAACGTGATTCCAACTGG
CX43	ACAGCGGTTGAGTCAGCTTG	GAGAGATGGGGAAGGACTTGT
Trpm7	AGGATGTCAGATTTGTCAGCAAC	CCTGGTTAAAGTGTTCACCCAA
c-Kit	GCCACGTCTCAGCCATCTG	GTCGCCAGCTTCAACTATTAACT

**Table 2:** HPLC elution protocol.

Number	Time	A	B
1	0 min	95%	5%
2	5 min	95%	5%
3	7 min	80%	20%
4	8 min	95%	5%
5	10 min	95%	5%

**Table 3:** Parameter settings in MRM mode.

Number	Compounds	MW	MRM Transitions	Cone (V)	Collision (V)	Ion Mode
1	Gly-Pro	172.1	173.0 > 116.0	28	14	ES+
2	Hyp-Gly	188.1	189.1 > 85.9	20	18	ES+
3	Pro-Hyp	228.2	229.2 > 69.9	30	16	ES+
4	Glu-Hyp	260.2	261.2 > 243.2	25	15	ES+
5	Ser-Hyp-Gly	275.2	276.1 > 189.0	28	16	ES+
6	Gly-Pro-Hyp	285.1	286.2 > 127.2	32	16	ES+

## Data Availability

The original contributions presented in this study are included in the article. Further inquiries can be directed to the corresponding author.
